# The PIM inhibitor AZD1208 synergizes with ruxolitinib to induce apoptosis of ruxolitinib sensitive and resistant JAK2-V617F-driven cells and inhibit colony formation of primary MPN cells

**DOI:** 10.18632/oncotarget.5653

**Published:** 2015-10-12

**Authors:** Lucia Mazzacurati, Que T. Lambert, Anuradha Pradhan, Lori N. Griner, Dennis Huszar, Gary W. Reuther

**Affiliations:** ^1^ Department of Molecular Oncology, Moffitt Cancer Center, Tampa, FL, USA; ^2^ Oncology iMed, AstraZeneca, Waltham, MA, USA

**Keywords:** MPN, JAK2-V617F, therapy, PIM, kinase inhibitor

## Abstract

Classical myeloproliferative neoplasms (MPNs) are hematopoietic stem cell disorders that exhibit excess mature myeloid cells, bone marrow fibrosis, and risk of leukemic transformation. Aberrant JAK2 signaling plays an etiological role in MPN formation. Because neoplastic cells in patients are largely insensitive to current anti-JAK2 therapies, effective therapies remain needed. Members of the PIM family of serine/threonine kinases are induced by JAK/STAT signaling, regulate hematopoietic stem cell growth, protect hematopoietic cells from apoptosis, and exhibit hematopoietic cell transforming properties. We hypothesized that PIM kinases may offer a therapeutic target for MPNs. We treated JAK2-V617F-dependent MPN model cells as well as primary MPN patient cells with the PIM kinase inhibitors SGI-1776 and AZD1208 and the JAK2 inhibitor ruxolitinib. While MPN model cells were rather insensitive to PIM inhibitors, combination of PIM inhibitors with ruxolitinib led to a synergistic effect on MPN cell growth due to enhanced apoptosis. Importantly, PIM inhibitor mono-therapy inhibited, and AZD1208/ruxolitinib combination therapy synergistically suppressed, colony formation of primary MPN cells. Enhanced apoptosis by combination therapy was associated with activation of BAD, inhibition of downstream components of the mTOR pathway, including p70S6K and S6 protein, and activation of 4EBP1. Importantly, PIM inhibitors re-sensitized ruxolitinib-resistant MPN cells to ruxolitinib by inducing apoptosis. Finally, exogenous expression of PIM1 induced ruxolitinib resistance in MPN model cells. These data indicate that PIMs may play a role in MPNs and that combining PIM and JAK2 kinase inhibitors may offer a more efficacious therapeutic approach for MPNs over JAK2 inhibitor mono-therapy.

## INTRODUCTION

Myeloproliferative neoplasms were first recognized in 1951 and today, classical Philadelphia chromosome-negative MPNs include polycythemia vera (PV), essential thrombocythemia (ET), and primary myelofibrosis (PMF) [1, 2]. These neoplasms are clonal hematopoietic stem/progenitor cell diseases that lead to a combination of erythrocytosis, thrombocytosis, leukocytosis, and scarring of the bone marrow [3]. Myelofibrosis patients also have an elevated risk of developing acute myeloid leukemia. Traditional therapies have included chemotherapy, phlebotomy, and other palliative approaches to relieve patient symptoms. Other than stem cell transplantation, which is not an option for most patients, there is no curative therapy for MPNs [4].

Classical MPNs are driven by aberrant JAK2 activation and signaling, presumably through known JAK2 effector pathways including STAT5, ERK, and Akt [5]. The *JAK2-V617F* activating mutation is observed in nearly all cases of PV and about half of the cases of ET and PMF [6]. In addition to JAK2-V617F, mutations in exon 12 of *JAK2* as well as JAK2 activating mutations in other signaling proteins, such as Mpl and Lnk, are found in MPNs [6–9]. *CalR* mutations are found in the majority of MPN patients that do not contain a *JAK2* or *Mpl* mutation [10]. While the ability of mutant CalR to activate STAT5 signaling is not completely clear, such cells do express a gene expression profile consistent with activation of the JAK2-STAT5 pathway as in JAK2-mutant positive MPNs [11]. While this genetic data alone suggests JAK2 activation plays an etiologic role in MPNs, a plethora of mouse models have demonstrated that expression of JAK2-V617F, as well as other JAK2-activating mutations found in MPNs, can generate human MPN-like phenotypes in mice [8, 9, 12–19].

The JAK1/2 inhibitor ruxolitinib was approved for some myelofibrosis patients in 2011 and for hydroxyurea resistant or intolerant PV patients in 2014 [20]. However, ruxolitinib, like other clinically tested JAK2 inhibitors, is unable to appreciably reduce allele burden in patients and thus does not induce remission. However, it does reduce constitutional symptoms associated with the disease, an effect believed to be due to the ability of the drug to inhibit JAK1 activation in the cytokine storm that is associated with MPNs [21]. Importantly, it was reported that ruxolitinib treatment might increase survival in high-risk myelofibrosis patients [22–24]. Nonetheless, it became evident that the neoplastic cells of MPN patients quickly developed resistance to JAK2 inhibitors.

Because JAK2 signaling is not suppressed long term and molecular remission is not observed in patients treated with JAK2 inhibitors, combination therapies have been investigated. Such combinations include JAK2 inhibitors with other signaling inhibitors such as inhibitors of PI3K/Akt and mTOR [25–29], as well as with drugs that decrease JAK2 expression and thus sensitize cells to JAK2 inhibition [30–33]. STAT5 is required for JAK2-V617F-induced MPN in mice [34, 35], and a JAK/STAT gene expression signature is observed in MPNs [11]. These data suggest STAT5 transcriptional targets play a role in MPNs and thus provide possible targets for therapeutic intervention.

Members of the *PIM* family of proto-oncogenes are STAT transcriptional targets [36–39]. PIMs are serine threonine kinases that cooperate with cMyc to induce lymphomagenesis in mice [40–43]. The anti-apoptotic signaling activity of PIMs likely contributes to their transforming activity [38, 44, 45]. PIMs are constitutively active kinases, possibly because of the unique kinase domain hinge region [46]. Thus, PIM activity is regulated via protein expression through transcriptional activation (*e.g*. JAK/STAT signaling) and regulation of protein turnover [42, 44, 46, 47]. Recent work has determined that PIM1 plays a role in hematopoietic stem cell growth and viability [48]. This is presumably through downstream pathways regulated by PIM kinase activity, which include, among others, the apoptotic activity of BAD and regulation of the mTOR pathway [42, 47]. PIM family members are therapeutic targets to consider in MPNs for numerous reasons, including their regulation of expression/activity by JAK2/STAT signaling, their ability to regulate hematopoietic stem cell growth, and their ability to inhibit apoptosis and function as hematopoietic oncogenes. Importantly, JAK2-V617F cannot induce PIM1 expression or an MPN phenotype in mice lacking STAT5 [35]. Finally, the lack of significant phenotype in PIM triple knockout mice suggests specific targeting of PIMs may not have overt adverse effects. Indeed, PIM inhibitors have demonstrated effectiveness as a therapeutic in a number of types of cancer models, including models of various hematopoietic cancers [49–55].

In this study we utilized a highly specific and effective PIM inhibitor, AZD1208 [52], and determined its effect on MPN cells alone and in combination with the JAK2 inhibitor ruxolitinib. Our data suggest primary MPN patient cells are sensitive to AZD1208 mono-therapy while AZD1208 mono-therapy has limited effect on MPN model cell lines. In combination with ruxolitinib, however, AZD1208 synergistically enhances the inhibitory effect of the JAK2 inhibitor in both MPN model cells and primary cells from MPN patients. In addition, AZD1208 can re-sensitize ruxolitinib resistant cells to undergo apoptosis in the presence of the JAK2 inhibitor and exogenous PIM1 expression can induce ruxolitinib resistance in JAK2 or Mpl driven cells. These data suggest targeting PIMs may enhance the efficacy of JAK2 inhibitors in MPNs.

## RESULTS

### PIM inhibitors have little effect on MPN model cell lines

**Figure 1:**
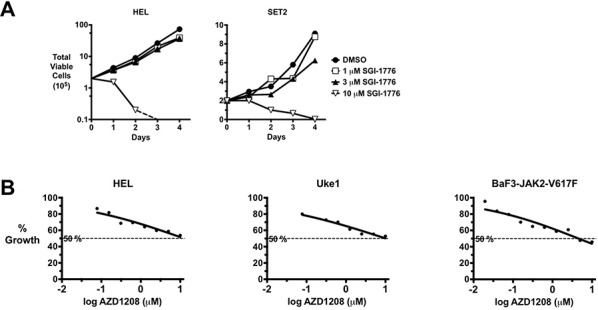
PIM Inhibitors lack significant efficacy against MPN model cells **A.** The MPN model/JAK2-V617F-expressing cell lines HEL and SET2 were cultured with the indicated concentrations of the PIM inhibitor SGI-1776. Total viable cells were determined over time using trypan blue exclusion. **B.** The MPN/JAK2-V617F-expressing cells HEL, Uke1, and BaF3-JAK2-V617F were treated with a range of concentrations of the PIM inhibitor AZD1208 and relative viable cells were determined by MTS assay. Percent growth relative to DMSO control is plotted versus the log of AZD1208 concentration. Fifty percent growth/inhibition is indicated by a dashed line.

To begin investigating the role of PIMs in signaling by JAK2-V617F, we treated MPN model cells with the pan PIM inhibitor SGI-1776 [56]. Even at single digit micromolar doses, SGI-1776 had little effect on the growth of MPN model cells, including JAK2-V617F-positive HEL and SET2 cells (Fig. 1A). However, high dose of SGI-1776 (10 μM) proved very toxic to these cells (Fig. 1A). Given the high dose required and the fact that SGI-1776 is also a potent inhibitor of TrkA and Flt3 [56], we wanted to utilize a more effective and selective PIM kinase inhibitor. AZD1208 is a recently developed, highly effective, pan-PIM kinase inhibitor [52]. The IC_50_ of AZD1208 for PIM1, PIM2, and PIM3 is 0.4 nM, 5.0 nM, and 1.9 nM respectively, values that are over an order of a magnitude improved compared to that reported for SGI-1776 [52, 56]. After PIMs, the kinase with the next highest affinity for AZD1208 has a binding constant that is over 43-fold higher than observed for PIMs [52]. The IC_50_ of AZD1208 for PIM1, PIM2, and PIM3 in cells was determined to be 10 nM, 151 nM, and 102 nM, respectively [52]. Thus, AZD1208 is a highly selective and efficacious PIM inhibitor. We treated MPN model cell lines, including JAK2-V617F-positive HEL, Uke1, and SET2 cells, and BAF3 cells that are transformed by the expression of JAK2-V617F (BaF3-JAK2-V617F), with AZD1208 and again saw little effect on cell growth and viability (Fig. 1B and not shown). In fact, the dose response curve for AZD1208 in these MPN model cells, which are routinely used to test MPN therapeutics, was not a classical sigmoidal shape, but rather was nearly linear (Fig. 1B). As seen in Fig. 1B, the IC_50_s of AZD1208 for MPN model cell lines were approximately 10 μM. This suggested that perhaps PIMs may not play a critical role in the growth of these MPN cell lines, which are dependent on signaling by JAK2-V617F.

### PIM inhibitors synergize with JAK2 inhibition against MPN cell growth and viability

**Figure 2:**
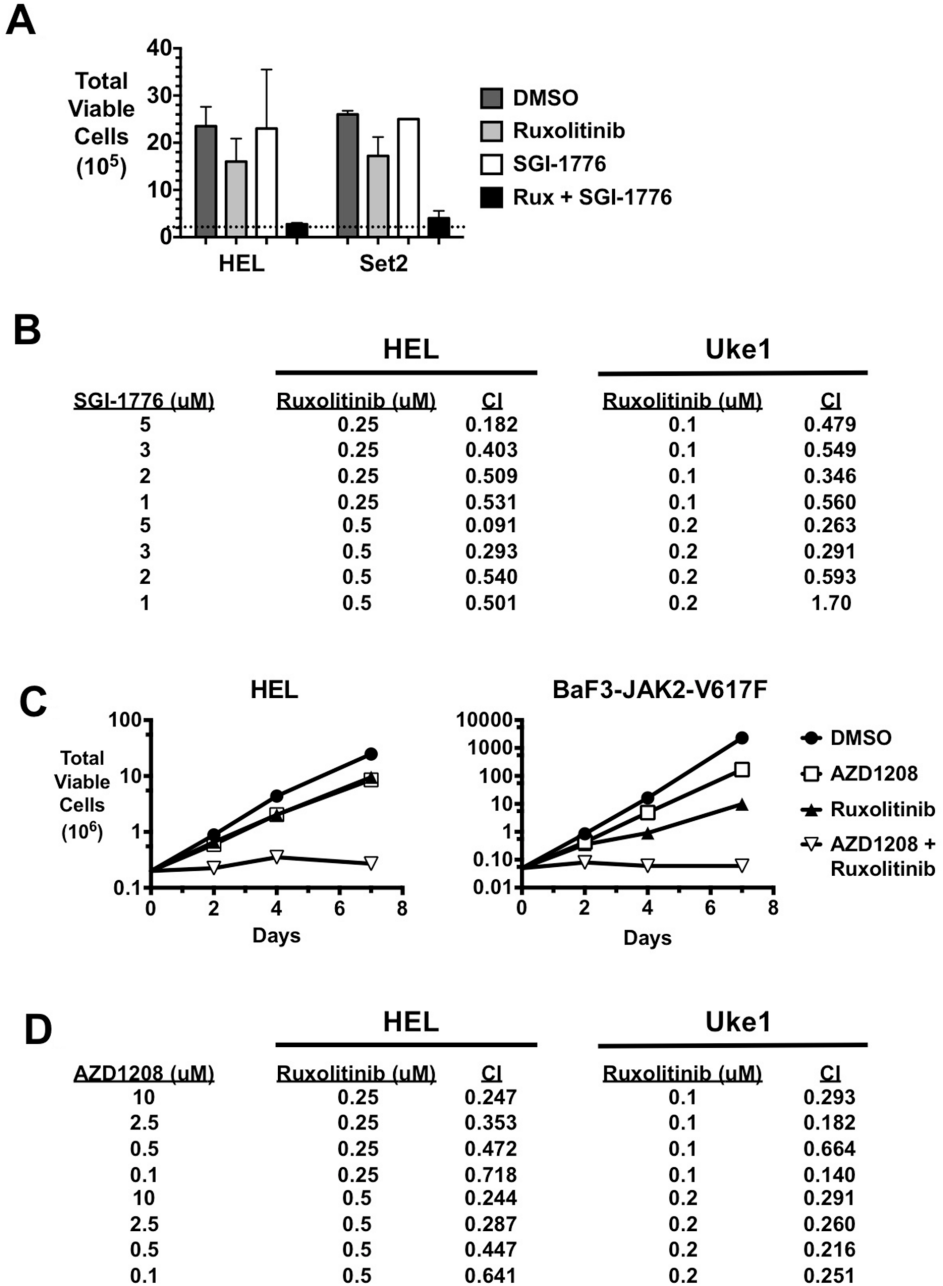
PIM inhibitors synergistically enhance the effect of ruxolitinib on the growth of MPN cells **A.** HEL and SET2 cells were cultured with DMSO, the JAK2 inhibitor ruxolitinib (Rux) (0.5 μM for HEL, 0.1 μM for SET2), the PIM kinase inhibitor SGI-1776 (3 μM), and the same concentrations of SGI-1776 and Rux in combination. Total viable cells were determined by trypan blue exclusion over time. The data shown represent the total number of HEL cells after three days of treatment and the total number of SET2 cells after ten days of treatment. The dashed line indicates the starting number of cells (2 × 10^5^) and error bars indicate standard deviation. **B.** HEL and Uke1 cells were treated with the indicated concentrations of SGI-1776 and ruxolitinib and relative viable cell number was determined by MTS assay. The percent inhibition of drugs alone and in combination was determined and the combination index (CI) for each combination was determined by Compusyn (Combosyn, Inc.). A combination index less than 1 indicates the combination therapy demonstrated synergy compared to the same concentrations of drugs used in mono-therapy treatment. **C.** HEL and BaF3-JAK2-V617F cells were treated with DMSO, AZD1208 (3 μM for HEL and 0.3 μM for BaF3-JAK2-V617F), ruxolitinib (0.25 μM for HEL and 0.1 μM for BaF3-JAK2-V617F), and AZD1208 plus ruxolitinib in combination. Total viable cells were determined over time by trypan blue exclusion. **D.** HEL and Uke1 cells were treated with the indicated concentrations of AZD1208 and ruxolitinib and relative viable cell numbers were determined by MTS assay. The percent inhibition of drugs alone and in combination was determined and the combination index (CI) for each combination was determined by Compusyn (Combosyn, Inc.).

While PIM inhibitors alone did not affect JAK2-V617F-driven growth, we wanted to determine if PIM inhibition could affect the extent of cell growth inhibition elicited by a JAK2 inhibitor. In this combination therapy, we observed synergistic effects on cell growth when the JAK2 inhibitor ruxolitinib was combined with either SGI-1776 (Fig. 2A & 2B) or AZD1208 (Fig. 2C & 2D) PIM inhibitors. In HEL and SET2 cells combination treatment effectively prevented the growth of the culture of cells over the course of the experiment (HEL: 3 days, SET2: 10 days) (Fig. 2A). While synergy was apparent in the data, formal synergy analyses demonstrated that this combination therapy indeed resulted in synergy (combination index less than 1) in both HEL and Uke1 cells (Fig. 2B) in all combinations of drug concentrations tested, with the exception of one combination in Uke1 cells. A combination of AZD1208 with ruxolitinib also effectively prevented the growth of MPN model cell lines, including HEL and BAF3-JAK2-V617F cells (Fig. 2C). Similar results were observed with SET2 cells treated with the inhibitors (data not shown). Again, combination indices of less than one were obtained when these two drugs were combined and used against both HEL and Uke1 cells (Fig. 2D). This demonstrated combining these two drugs also has a synergistic effect against MPN model cell lines. Importantly, combining ruxolitinib and SGI-1776 did not affect the growth of K562 cells, a myeloid cell line that's growth is not driven by JAK2-V617F but rather by BCR-ABL (not shown) and ruxolitinib and AZD1208 either in mono-therapy or in combination did not have any effect on Jurkat T-cell leukemia cells (not shown). This suggests that the combination of ruxolitinib and PIM inhibitors does not have a non-specific effect on cell growth.

### PIM inhibitors synergize with JAK2 inhibition to induce apoptotic cell death

**Figure 3:**
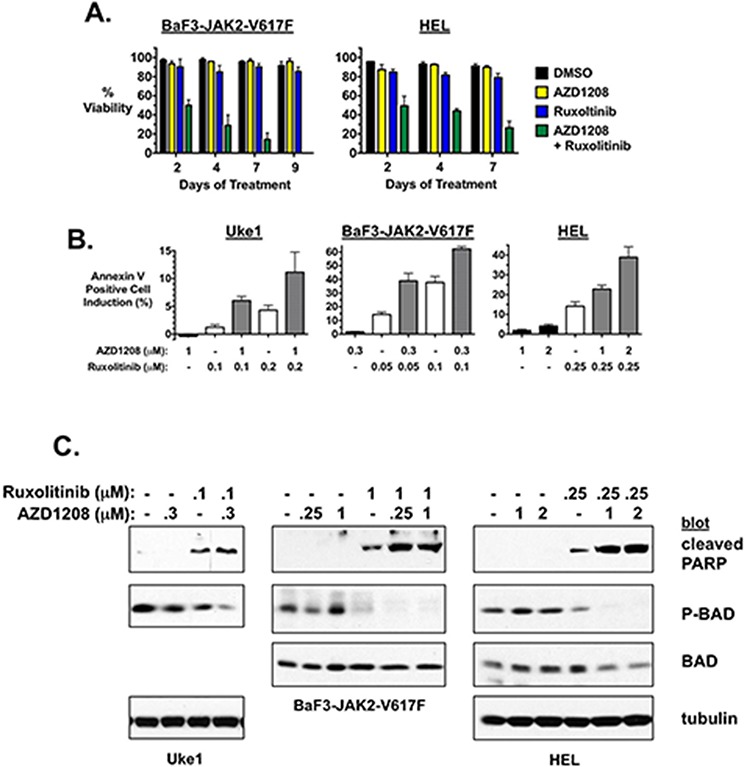
AZD1208 enhances apoptosis induced by ruxolitinib **A.** BaF3-JAK2-V617F and HEL cells were treated with DMSO, AZD1208 (0.3 μM for BaF3-JAK2-V617F and 3 μM for HEL), ruxolitinib (0.1 μM for BaF3-JAK2-V617F and 0.25 μM for HEL), and AZD1208 plus ruxolitinib in combination. Percent viability over time was determined by trypan blue exclusion. Error bars indicate standard deviation. **B.** The MPN model cell lines Uke1, BaF3-JAK2-V617F, and HEL were treated with DMSO, and the indicated concentrations of AZD1208 and ruxolitinib alone and in combination. Annexin V binding was determined by flow cytometry after 72 hours for Uke1, 48 hours for BaF3-JAK2-V617F, and 48 hours for HEL. Data is represented as the increase in the percent of annexin V positive cells compared to identically treated DMSO-treated cells. Error bars indicate standard deviation of samples treated in triplicate. **C.** Uke1, BaF3-JAK2-V617F, and HEL cells were treated with DMSO, AZD1208, and/or ruxolitinib, as indicated. Cell lysates were prepared after 24 hours (Uke1 and HEL) or 48 hours (BaF3) of treatment and immunoblots were performed for cleaved PARP, P-BAD (Ser-112), and tubulin (Uke1 and HEL) and/or total BAD (BaF3 and HEL) as controls, as indicated. Note: drug treatment did not alter total BAD expression in Uke1 cells (Supplementary Fig. 1C).

The mechanism by which combination of PIM and JAK2 inhibition inhibited cell growth appeared to be induction of cell death. Combination of AZD1208 and ruxolitinib induced augmented loss of cell viability over time compared to either drug alone at the concentrations used, as seen in BaF3-JAK2-V617F and HEL cells (Fig. 3A). These results were also observed in SET2 cells treated with the inhibitors (data not shown). In addition, combining the two drugs resulted in enhanced apoptotic cell death as determined by annexin V binding (Fig. 3B). While AZD1208 had no effect on cell death at the concentrations utilized, ruxolitinib alone and in a dose dependent manner enhanced the percent of the cell population that was undergoing apoptosis, as expected (Fig. 3B). However, the number of cells undergoing apoptosis was further induced by the presence of AZD1208, even though alone it did not induce significant cell death, resulting in a synergistic response (Fig. 3B). Similar results were obtained in all JAK2-V617F-dependent MPN model cell lines tested, including Uke1, BaF3-JAK2-V617F, and HEL (Fig. 3B) and SET2 (Supplementary Fig. 1A). PARP cleavage correlated with enhanced annexin binding in Uke1, BAF3-JAK2-V617F, and HEL cells (Fig. 3C) and SET2 cells (Supplementary Fig. 1B), confirming combined ruxolitinib and AZD1208 treatment led to enhanced apoptotic cell death compared to ruxolitinib treatment alone. A known PIM substrate is the pro-apoptotic protein BAD [42, 44, 45, 47]. Because PIMs are known to inactivate BAD by phosphorylation we analyzed the status of BAD phosphorylation in drug treated cells. A decrease in serine-112 phosphorylated BAD, which was not due to a decrease in BAD protein, was observed concomitant with an increase in the presence of cleaved PARP (Fig. 3C and Supplementary Fig. 1B). In all three lines tested BAD serine-112 phosphorylation was decreased the most by combination of ruxolitinib and AZD1208, corresponding to the enhanced apoptosis detected in cells treated with the combination of drugs (Fig. 3C and Supplementary Fig. 1A and 1B). Finally, to determine if combining ruxolitinib and AZD1208 affected the growth rate by affecting the cell cycle in addition to inducing apoptosis, we analyzed the cell cycle in treated cells. Combination treatment had no effect on the percent of cells in each phase of the cell cycle compared to ruxolitinib treated cells (data not shown). Together these data suggest the combinatorial effect on cell growth and viability that is observed with concomitant treatment with JAK2 and PIM inhibitors is due to enhanced induction of apoptotic cell death.

### PIM inhibitors inhibit erythropoietin-independent colony formation of primary MPN cells

**Figure 4:**
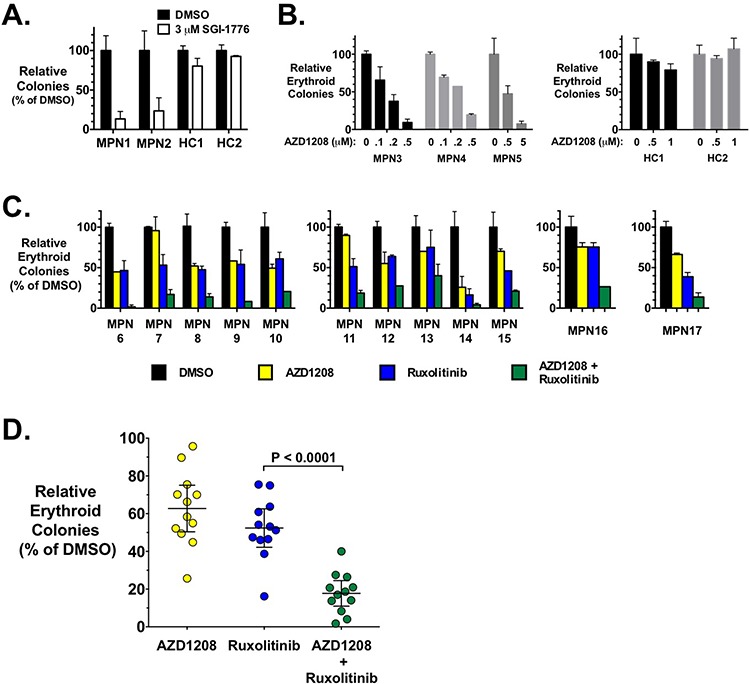
MPN patient erythroid colony formation is inhibited by AZD1208 mono-therapy and synergistically inhibited with AZD1208 and ruxolitinib combination therapy **A.** Peripheral blood mononuclear cells (PBMCs) from two MPN patients were plated in methylcellulose, containing cytokines but lacking erythropoietin (Epo), in the presence of DMSO or SGI-1776 (3 μM). Epo-independent erythroid colonies (EECs) were counted 14 days later. Similarly, cells from two healthy controls (HC) were plated in the same medium containing Epo, and erythroid colonies were determined 14 days later. Data are represented as percent of DMSO samples. **B.** PBMCs from three MPN patients (left) and two healthy controls (right) were plated, as in A., with the indicated doses of AZD1208. Erthyroid colonies were determined 14 days later and are represented as percent of DMSO samples. **C.** PBMCs from MPN patients were plated as in A. with DMSO, AZD1208, and ruxolitinib alone or in combination. Drug concentrations used: MPN6–10, 0.2 μM AZD1208 and 0.05 μM ruxolitinib; MPN11–15, 0.1 μM AZD1208 and 0.05 μM ruxolitinib; MPN16, 0.1 μM AZD1208, 0.01 μM ruxolitinib; and MPN17, 0.2 μM AZD1208 and 0.1 μM ruxolitinib. Erythyroid colonies were determined 14 days later and are represented as percent of DMSO samples. Error bars indicate standard deviation. **D.** Summary of data in C. with mean +/− 95% confidence interval indicated. *P* value was calculated by paired *t*-test. All samples were from JAK2-V617F-positive MPN patients: samples MPN1, 6, 7, 8, 12, 13, 15, and 16 were from PV patients; MPN3, 5, 9, 10, 14, and 17 were from ET patients; and MPN2, 4, and 11 were from MF patients.

In order to test the effect of PIM inhibition on primary cells from MPN patients, we utilized the characteristic ability of MPN progenitor cells to form erythropoietin-independent erythroid colonies (EEC) in methylcellulose [57, 58]. SGI-1776 inhibited EEC formation of the two MPN patient (both JAK2-V617F-positive) samples tested (Fig. 4A). Likewise, AZD1208 also inhibited EEC formation of cells from three additional JAK2-V617F-positive MPN patients and in a dose dependent manner (Fig. 4B) (see legend of Fig. 4 for more details). Interestingly, this is unlike what we observed in cell lines where PIM inhibitors alone were relatively ineffective (Fig. 1). Treatment of PBMCs from healthy controls showed no inhibition of erythroid colony formation by either SGI-1776 (Fig. 4A) or AZD1208 (Fig. 4B).

While JAK2 inhibitors inhibit EEC formation of primary MPN progenitor cells, we next tested the ability of PIM inhibition to augment the effect of JAK2 inhibition of EEC colony formation. To do this we utilized concentrations of both AZD1208 and ruxolitinib that would each elicit about 50% inhibition of colony growth. Thus, for these experiments we utilized 100 or 200 nM AZD1208 and 10–100 nM ruxolitinib, with most samples being treated with 100 or 200 nM AZD1208 and 50 nM ruxolitinib. Combining these two drugs resulted in an augmented effect on the inhibition of EEC formation of PBMCs isolated from MPN patients. Fig. 4C shows the effect of AZD1208 and ruxolitinib, alone and in combination, on EEC formation of primary cells from twelve JAK2-V617F-positive MPN patients. The response to drugs was somewhat variable, as expected with primary cells from different patients, but in all cases tested we observed a significant enhancement of growth inhibition, and in many cases a synergistic response, by the combination of ruxolitinib and AZD1208 (Fig. 4C). Comparison of the amount of inhibition of colony formation induced by JAK2 inhibition to the combination treatment in all samples tested, demonstrated that the combination treatment induced a statistically significant enhancement of inhibition of primary MPN cell colony formation (Fig. 4D).

### Combination of PIM and JAK2 inhibitors enhances dephosphorylation of proteins of the mTOR pathway

**Figure 5:**
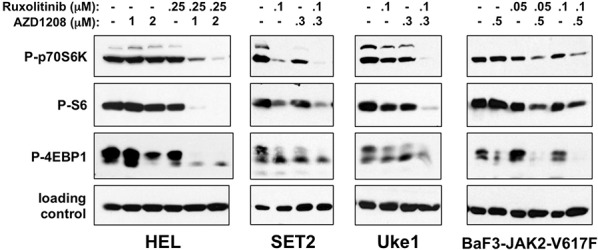
AZD1208 and ruxolitinib suppress downstream signaling of the mTOR pathway HEL, SET2, Uke1, and BaF3-JAK2-V617F cells were treated with DMSO (−) or the indicated amounts of AZD1208 and ruxolitinib, alone and in combination. Lysates were prepared following 24 hours for HEL cells, 4 hours for SET2 and Uke1 cells, and 72 hours for BaF3-JAK2-V617F cells. Lysates were analyzed by immunoblotting for P-p70S6K (T389), P-S6 (S235/236), P-4EBP1 (T37/46), and as loading controls tubulin (for HEL and Uke1) and GAPDH (for SET2 and BaF3-JAK2-V617F). Note: the loading control blot for HEL cells in this Fig. is the same as shown in Fig. 3, as the same lysates were analyzed in each Fig.

AZD1208 treatment of MPN cells frequently led to an increase in PIM1, PIM2, and PIM3 protein (Supplementary Fig. 2A and not shown), suggesting on target inhibition and subsequent stabilization of the protein, as previously reported [54, 59]. It has been previously shown that AZD1208 treatment targets proteins of the mTOR pathway that regulate protein translation [52]. Keeton et al. demonstrated that in AML, AZD1208, in addition to inhibiting BAD phosphorylation, also leads to decreased phosphorylation of p70S6K, ribosomal S6 protein, and 4EBP1 [52]. To investigate the effects of AZD1208 on these proteins at doses that induced enhanced apoptosis with combination of AZD1208 and ruxolitinib, we treated cells utilizing similar drug doses and followed with immunoblot analyses. At the doses utilized we observed primarily subtle and varied decreases in phosphorylation of p70S6K (Thr-389) in all four MPN cell lines utilized (Fig. 5). However, in all cell lines combination of the two drugs led to the most significant decreases in p70S6K phosphorylation. Correlating with this was a loss in the phosphorylation of ribosomal protein S6 (Ser-235 and Ser-236). Again we observed slight decreases in phosphorylated S6 when drugs were used at these concentrations alone. Maximal effect was again observed upon co-treatment with AZD1208 and ruxolitinib. Finally, we observed inhibition of phosphorylation of 4EBP1 (Thr-37/Thr-46) by both AZD1208 and ruxolitinib. While we utilized an antibody that recognizes phosphorylated Thr-37 and/or Thr-46 of 4EBP1, we also observed an increase in the mobility of 4EBP1 that is indicative of the loss of hyperphosphorylation of 4EBP1 (Fig. 5 and Supplementary Fig. 2A). Again the combination of AZD1208 and ruxolitinib provided maximally observed decreases in 4EBP1 phosphorylation. The decreases in phosphorylation of p70S6K, S6, and 4EBP1, which were not due to a decrease in levels of these proteins (Supplementary Fig. 2A and 2B), suggest that combining AZD1208 and ruxolitinib enhances inhibition of downstream components of the mTOR pathway. It should be noted that while PIM protein levels can be controlled by JAK2/STAT5 signaling, the low doses of ruxolitinib utilized to demonstrate combinatorial effects do not completely eliminate PIM protein expression (Supplementary Fig. 2A). Finally, we observed similar effects of AZD1208 and ruxolitinib on phosphorylation of p70S6K and S6 in primary granulocytes from a PV patient (Supplementary Fig. 3).

### PIM inhibition sensitizes JAK2 inhibitor-resistant cells to JAK2 inhibition

**Figure 6:**
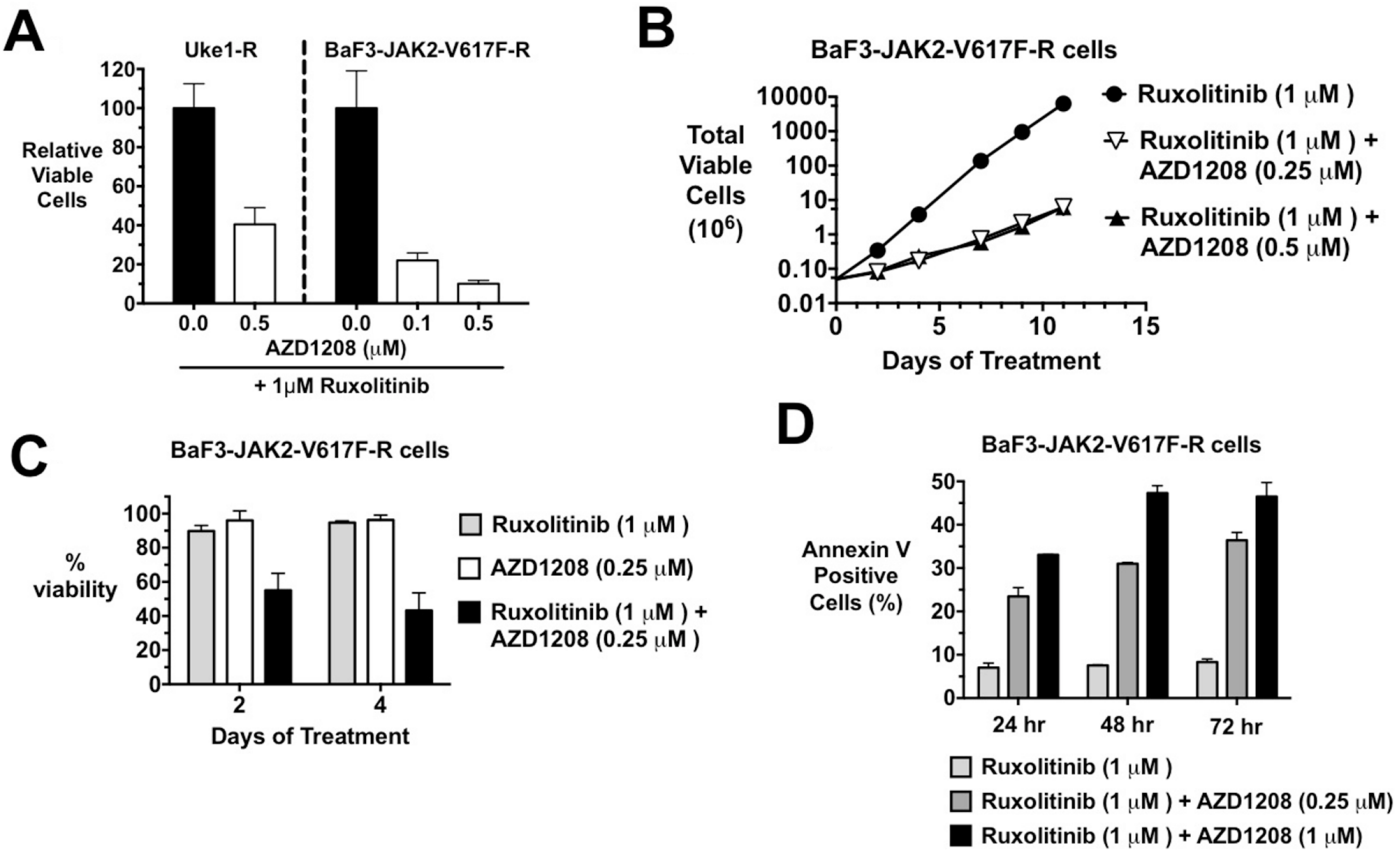
MPN cells that persistently grow in the presence of JAK2 inhibitors are still sensitive to the combination of ruxolitinib and AZD1208 **A.** Ruxolitinib persistent Uke1-R and BaF3-JAK2-V617F-R cells growing in 1 μM ruxolitinib were plated in 1 μM ruxolitinib and 0.1 or 0.5 μM AZD1208, as indicated. Relative viable cells were determined by MTS assay after 72 hours. **B.** Ruxolitinib persistent BaF3-JAK2-V617F-R cells were cultured in 1 μM ruxolitinib alone or with 0.25 or 0.5 μM AZD1208 and total viable cells were determined over time by trypan blue exclusion. **C.** BaF3-JAK2-V617F-R cells growing in 1 μM ruxolitinib were treated with ruxolitinib alone, 0.25 μM AZD1208, or the combination of the two drugs. Cell viability after two and four days was determined by trypan blue exclusion. **D.** Apoptosis in BaF3-JAK2-V617F-R cells treated with 1 μM ruxolitinib, 0.25 μM AZD1208, or a combination of the two drugs was detected with annexin V staining and flow cytometry after 24, 48, and 72 hours. Error bars indicate standard deviation.

We have derived JAK2-V617F-dependent MPN cells to be resistant to JAK2 inhibition (also referred to as persistent cells) by chronic, dose-escalation exposure to ruxolitinib, as previously described [60]. JAK2 mutations were not selected for (or detected) in these cells, consistent with Koppikar et al. [60]. Because PIM1 and 2 are regulated by STAT5 activation, and thus are downstream effectors of JAK2 signaling, we checked the expression of all three PIM family members in these cells that are able to grow in the presence of high levels of JAK2 inhibitor it is redundant and shouldn't be there and compared expression levels to ruxolitinib sensitive cells. In general, and as expected, both PIM1 and PIM 2 mRNA levels were sensitive to ruxolitinib treatment in ruxolitinib sensitive cells. This was observed in all three lines tested, including HEL, SET2, and Uke1 (Supplementary Fig. 4A). PIM3 expression, however, was less responsive to ruxolitinib, as inconsistent decreases were observed. In ruxolitinib persistent cells growing in the chronic presence of the drug, PIM mRNA levels were elevated compared to the levels observed with acute downregulation in drug sensitive cells (Supplementary Fig. 4A). In all cell lines, mRNA levels of two PIM family members reached 50% to nearly 100% that observed as steady state levels of drug sensitive cells. PIM1 and PIM2 protein levels were decreased by acute high dose ruxolitinib treatment, while PIM3 protein was not significantly affected (Supplementary Fig. 4B). Expression of all three PIM family members was readily observed in ruxolitinib persistent cells growing in 1 μM ruxolitinib (Supplementary Fig. 4B and Supplementary Fig. 6), where PIM protein levels were equal to or greater than control/uninhibited levels (Supplementary Fig. 4B). This suggested that the expression of PIMs may play a role in the ruxolitinib resistant state. To test this we treated ruxolitinib persistent cells, which were continuously growing in the presence of ruxolitinib, with AZD1208. AZD1208 treatment of ruxolitinib persistent Uke1 (Uke1-R) and BaF3-JAK2-V617F-R cells growing in 1 μM of ruxolitinib resulted in growth inhibition (Fig. 6A) in a short term MTS assay. Similar results were obtained with SET2-R and HEL-R cells (not shown) as well as with the PIM inhibitor SGI-1776 (Supplementary Fig. 5). To investigate longer-term growth we treated ruxolitinib persistent BaF3-JAK2-V617F-R cells with AZD1208. Over an eleven-day time course, persistent cells growing in 1 μM ruxolitinib increased in cell number about 1000-fold that of the same cells treated with AZD1208 (Fig. 6B). Qualitatively similar results were obtained with ruxolitinib persistent Uke1-R cells (not shown). AZD1208 treatment of ruxolitinib persistent BaF3-JAK2-V617F-R cells resulted in a substantial loss in cell viability (Fig. 6C) and this loss was due to a significant induction of apoptosis (Fig. 6D). These ruxolitinib-persistent cells growing in 1 μM ruxolitinib had a stable steady state level of annexin V positive cells (about 8%). However, the addition of AZD1208 to these cells enhanced the percent of annexin V positive cells to as high as 47% (Fig. 6D). AZD1208 alone had no effect on ruxolitinib persistent cells in the absence of the JAK2 inhibitor (Fig. 6C). Thus, cells exhibiting ruxolitinib persistent growth, and which maintain elevated PIM protein levels (Supplementary Fig. 4B and Supplementary Fig. 6), could be re-sensitized to apoptosis by the concomitant treatment with AZD1208. AZD1208 treatment of ruxolitinib persistent cells did not affect the activation state of JAK2 signaling effectors, suggesting AZD1208 is not altering JAK2 activation and signaling in these cells (Supplementary Fig. 6).

### PIM1 expression is sufficient to induce ruxolitinib resistance

**Figure 7:**
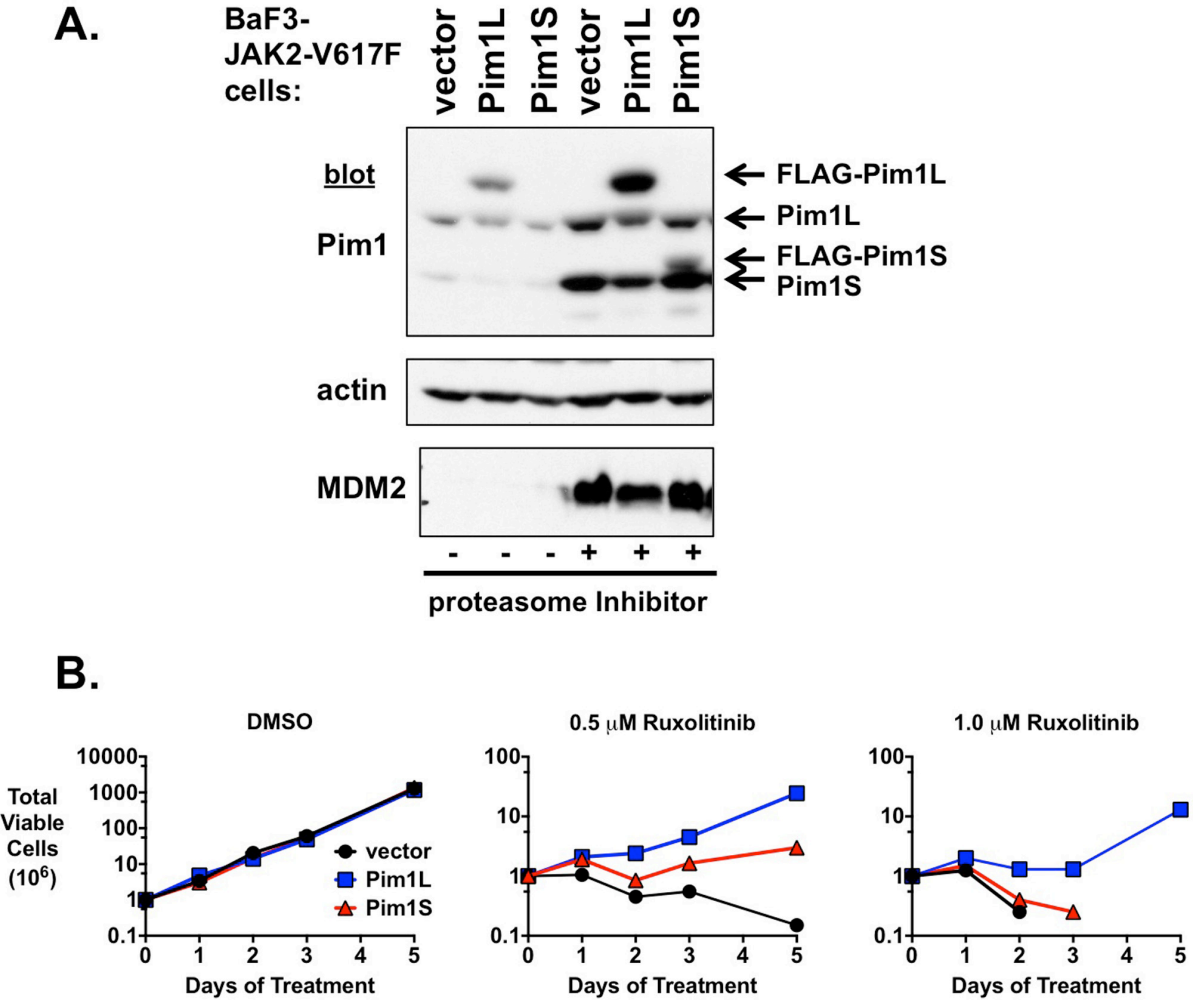
Exogenous expression of PIM1 induces ruxolitinib resistance **A.** PIM1L and PIM1S were expressed from a viral promoter in BaF3-JAK2-V617F cells. Cell lysates were immunoblotted for PIM1, actin as a loading control, and MDM2 for a proteasome inhibitor control. The proteasome inhibitor bortezomib was utilized to stabilize PIM1 expression for easier detection of PIM1S. The exogenous PIM1 proteins were FLAG-tagged thus increasing their molecular weight and slowing their mobility in SDS-PAGE compared to endogenous PIM1 proteins. Mobility of endogenous and exogenous PIM1 proteins are indicated with arrows. **B.** These cells from A., along with vector control, were cultured with DMSO or ruxolitinib (0.5 μM and 1.0 μM) and total viable cells were determined over time by trypan blue exclusion. Similar results were obtained in three independent experiments.

Our data suggest PIM inhibition synergizes with ruxolitinib to induce growth inhibition and apoptosis in MPN model cells. In addition, while JAK2 inhibition results in a decrease in PIM expression, this expression is augmented in cells that are resistant to JAK2 inhibitors (Supplementary Fig. 4B and Supplementary Fig. 6). Also, PIM proteins can function as oncogenes themselves under certain conditions. Taken together, we wanted to determine if aberrant PIM expression could induce JAK2 inhibitor resistance. To do this we expressed PIM1 in BaF3 cells transformed to cytokine independence by JAK2-V617F. These cells depend on activated JAK2 for growth, and our expression of PIM1 via a retroviral promoter would effectively uncouple the expression of PIM1 from the control of JAK2/STAT5 signaling. We expressed both the long and short forms of PIM1 in these experiments (Fig. 7A). PIM1L was expressed at a higher level than PIM1S, which could only be detected following proteasome treatment of cells (Fig. 7A). As expected, proteasome treatment increased both exogenous and endogenous PIM1 protein levels (Fig. 7A). Expression of PIM1 in BaF3-JAK2-V617F cells did not alter their rate of cytokine independent growth (Fig. 7B, first graph). While BaF3-JAK2-V617F cells expressing a control vector remain sensitive to the JAK2 inhibitor ruxolitinib, the expression of PIM1 in these cells led to an outgrowth of cells that could persistently grow in the presence of ruxolitinib (Fig. 7B, second and third graphs)). PIM1L was much more effective at inducing ruxolitinib resistance than PIM1S, presumably due to the higher level of exogenous PIM1L. PIM1L expression was able to induce resistance to 0.5 and 1.0 μM ruxolitinib, with continued proliferation of the cells. Of note, expression of PIM1 proteins in parental IL-3-dependent BaF3 cells did not induce cytokine independent growth (not shown), suggesting the outgrowth of PIM1-expressing JAK2-V617F-transformed cells was not due to the ability of exogenous PIM1 to transform these cells on its own. Exogenous PIM1 expression similarly induced ruxolitinib resistance in 32D myeloid cells transformed by the MPN oncogenic protein Mpl-W515L (Supplementary Fig. 7). These data suggest PIM proteins may be sufficient to induce JAK2 inhibitor resistance and the expression of PIM proteins may play a role, downstream of re-activated JAK2 signaling, in the development of JAK2 inhibitor resistance.

## DISCUSSION

The lack of efficacy of JAK2 inhibitors in patients is a major roadblock in the development of effective targeted therapies for MPNs. Because ineffective JAK2 inhibition leaves JAK2 signaling intact, combination therapies are actively being investigated. Heat shock protein inhibitors and histone deacetylase inhibitors destabilize JAK2 and thus render cells more sensitive to JAK2 inhibitors [30–33]. Combination therapies with agents that target signaling molecules downstream of JAK2 have also been investigated. Inhibitors of PI3K and mTOR, for example, have been shown to be effective in MPN models and in combination with JAK2 inhibitors [25–29].

In this report we demonstrate that PIM inhibitors synergize with JAK2 inhibitors against MPN cell growth and viability. We chose to target PIM kinases for numerous reasons, including: 1. members of the *PIM* family of genes are transcriptionally activated by JAK/STAT5 signaling [42, 46, 47]; 2. PIMs are constitutively active kinases regulated by expression through transcription and protein stability [42, 44, 46, 47]; 3. STAT5 is required for MPN formation in mouse models and PIM1 is not induced in such models in the absence of STAT5 [34, 35]; 4. *PIM* family members can function as hematopoietic oncogenes [40–43, 49]; and 5. PIM1 regulates hematopoietic stem cell growth [48].

Treatment of MPN cell lines with the PIM inhibitor AZD1208 was rather ineffective at blocking cell growth, with IC_50_ values of around 10 μM (Fig. 1). This is in contrast to numerous AML cell lines that are sensitive to AZD1208 treatment, suggesting the ability of AZD1208 to inhibit malignant myeloid cell growth may be dependent on factors such as the cellular driving mutation [52]. On the contrary, AZD1208 was much more effective at inhibiting erythropoietin independent erythroid colony formation of primary cells from MPN patients (Fig. 4). In this assay, 0.1 - 0.2 μM consistently inhibited about 50% of colony formation, whereas colonies from healthy controls were not inhibited at 1 μM. The ability of AZD1208 to block primary MPN cell colony formation and not MPN cell line growth may be due to the importance of PIMs in the proliferation of hematopoietic stem and progenitor cells [48], which are responsible for colony development in hematopoietic colony formation assays. It is possible AZD1208 may differentially target signaling pathways in primary cells compared to cell lines. Alternatively, the expression of PIM family proteins in erythroid progenitors may be lower than in cell lines, thus making such cells more sensitive to AZD1208. However, the lack of a significant dose-response effect in cell lines (Fig. 1), in particular at very high inhibitor doses, suggests PIM levels would likely not be the sole reason for this discrepancy in sensitivity to AZD1208. Complexity is added by the fact that AZD1208 is a pan-PIM inhibitor of the three PIM members [52]. It is quite possible that cell lines have different signaling than primary cells that may make them more resistant to PIM inhibition. For example, PIM and Akt substrates overlap and thus differential Akt activity in cell lines, compared to primary cells, could mask the effect of PIM inhibition. With this said, it is worth noting that our results suggest that experiments aimed at the development of potential MPN therapeutics should consider the use of primary samples, even in the face of negative results with MPN cell lines. In a more general sense, our results exemplify the potential to generate mis-leading/less accurate conclusions from data solely obtained using established cell lines.

*PIM1* knockout and *PIM1* transgenic expression inhibits and enhances hematopoiesis, respectively, providing evidence of a role for this serine threonine kinase in hematopoietic malignancies [48]. Because *PIM1* transgenic overexpression in mice enhances hematopoiesis, chronic PIM1 expression in MPNs by constitutive JAK2/STAT5 signaling may contribute to aberrant hematopoiesis in MPNs. As ruxolitinib mono-therapy is unable to reduce allele burden/induce remission in patients, a treatment strategy that increases neoplastic stem/progenitor cell death is needed. The ability of AZD1208 to enhance ruxolitinib-induced apoptosis of JAK2-V617F-driven cells is thus significant (Fig. 3). Using different small molecules, Huang *et al*. also demonstrated PIM inhibition enhances the efficacy of JAK2 inhibitor therapy [54]. Our work demonstrating that AZD1208 synergizes with ruxolitinib to inhibit primary MPN cell colony formation provides additional and significant pre-clinical data that PIM inhibition may increase the therapeutic efficacy of JAK2 inhibitors (Fig. 4).

PIM inhibitor treatment in different hematological disease models does not result in consistent effects on cell signaling, and thus PIMs may play different roles in different cancers. In chronic lymphocytic leukemia, the PIM inhibitor SGI-1776 induces apoptosis by decreasing Mcl1 express via a global block in RNA synthesis, and in multiple myeloma the mechanism of action is by blocking translation and inducing autophagy [55, 56]. However, SGI-1776 is not specific for PIMs as it also inhibits c-Kit and TrkA [56]. AZD1208 is a much more specific PIM inhibitor and in AML it was determined that AZD1208 primarily blocks phosphorylation of p70S6K and 4EBP1 resulting in inhibition of translation [52]. It should be noted that in order to demonstrate an augmented or synergistic effect of combining PIM and JAK2 inhibition to enhance the efficacy of targeting MPN cells, the dose of ruxolitinib we utilized in our studies was suboptimal for the effect under investigation (*e.g*. decrease in phosphorylation of biomarkers, apoptosis induction, etc.). Thus, while JAK2 inhibition in MPN cells can inhibit PIM expression (Supplementary Fig. 4B), low drug concentrations do not eliminate all PIM protein, which would remain active (Supplementary Fig. 2A), as would be the case for incomplete target inhibition of JAK2 inhibitors. With this said, it should be noted that PIM3 expression is refractory to high dose ruxolitinib treatment (Supplementary Fig. 4B). The decrease in phosphorylation of p70S6K, S6, and 4EBP1 was only consistently observed with AZD1208 in combination with ruxolitinib (Fig. 5). Phosphorylation of 4EBP1 prevents it from binding and inhibiting the translation initiation factor eIF4E [61]. Thus AZD1208 was shown to decrease cap-dependent translation in AML cells, an observation made with inhibiting PIMs in other cell types [62, 63]. Our similar observation of a decrease in phosphorylation of 4EBP1 as well as p70S6K and ribosomal protein S6, suggests AZD1208 may be inhibiting cap-dependent translation in JAK2-V617F-driven cells. Because the loss of phosphorylation of these regulators of translation is most prominent in cells treated with ruxolitinib and AZD1208 in combination, it is possible the combinatorial effects of these drugs may be due in part to inhibition of this mTOR regulated pathway. Again, this is supported by the synergy observed with JAK2 and mTOR inhibitors in MPN cells and the observations made by Huang et al. [54]. Thus, as cancer cells have been shown to be more addicted to protein translation than normal cells [64], eIF4E may be a potential therapeutic target for MPNs, a concept strongly supported by the recent determination that decreasing cellular eIF4E levels can inhibit cellular transformation *in vivo* without affecting normal development [65].

In addition to the observation, of ours and Huang et al. [54], that downstream components of the mTOR pathway in MPN cells are inhibited by PIM inhibitors, we also observed a decrease in phosphorylation of BAD at Ser-112, a known phosphorylation target of PIM. Since this phosphorylation inhibits the pro-apoptotic activity of BAD, decreased BAD phosphorylation would lead to activation of BAD and subsequent apoptosis [42, 44, 45, 47]. The decrease in BAD Ser-112 phosphorylation (Fig. 3C), along with the enhanced apoptosis (Fig. 3B) and lack of effect on cell cycle (not shown), are consistent with recent work that demonstrated that phosphorylation of BAD plays a key role downstream of JAK2 in MPN cell viability [66]. Thus, it is likely the decreased BAD phosphorylation in response to AZD1208/ruxolitinib combination therapy contributes to the enhancement of apoptosis induced by the same concentration of ruxolitinib alone (Fig. 3B). BAD phosphorylation at Ser-112 is also regulated by activated Akt and ERK, likely explaining the variable decrease in phosphorylated BAD by PIM inhibition alone (at the doses analyzed) (Fig. 3C). This is consistent with the concept proposed for the roles of different JAK2 effector pathways in the inactivation of BAD in MPN cells [66]. Unfortunately we have not been successful at knocking down PIM proteins by RNA interference approaches in our MPN cell lines to further test some of our results, but previous work demonstrated a small effect on the short term growth of HEL cells upon transient knockdown of PIM1 and PIM2 [67], and more recent work showed that targeting PIMs may sensitize MPN cells to JAK2 inhibitors through down regulation of c-Myc [54]. Taken together, these data suggest that PIMs play a role in JAK2-V617F-mediated neoplastic cell growth and may be a potential site for therapeutic targeting to enhance the efficacy of ruxolitinib.

The persistent growth of JAK2-driven cells in drug treated patients may be due to incomplete target inhibition by JAK2 inhibitors. While JAK2 mutations can render resistance to kinase inhibitors, such mutations have never been found in patients treated with JAK2 inhibitors [60, 68]. JAK2 heterodimerization with JAK1 and Tyk2 may provide a mechanism of persistent growth in the presence of JAK2 inhibitors, possibly through transactivation of the complexed JAK family members [60]. However, how JAK2 remains active in such complexes in the presence of the inhibitor, which also inhibits JAK1 in the case of ruxolitinib, is not known. JAK2 is still required for this persistent state of resistance suggesting such interactions with other JAK family members may preclude the ability of current inhibitors from accessing the ATP-binding site of JAK2, or that JAK2 is functioning as a scaffold with its kinase activity being compensated for by other complexed kinases. Additional mechanisms of resistance that have been proposed involve the activation of Ras effector pathways by mutated Ras or GNB1 [66, 69]. These include the ERK and AKT pathways, both of which are also effectors of activated JAK2. Thus, JAK2 inhibitor resistance may be maintained by alternative mechanisms of activation of downstream effectors.

JAK2-dependent MPN cells that are developed to be resistant to a JAK2 inhibitor are cross-resistant to other JAK2 inhibitors [60], suggesting MPN patients may be resilient to alternative JAK2 inhibitors following initial resistance. While this will likely be true for many patients, current clinical data does suggest alternative JAK2 inhibitors may improve symptoms in some patients that are resistant to ruxolitinib [70, 71]. In addition, more recently described type II inhibitors may be effective in such patients (see below). Nonetheless, combination therapies designed to target a key effector downstream of JAK2 may be more effective because not only might such therapies target JAK2 and augment the efficacy of JAK2 inhibitors, but such approaches may also render cells less susceptible to drug resistance because of continuous inhibition of JAK2 effector pathways. We demonstrate that ruxolitinib-persistent (resistant) JAK2-V617F-dependent cells are resensitized to the drug upon treatment with a PIM inhibitor (Fig. 6). Circumventing the control of PIM1 expression by the JAK2/STAT5 pathway by expressing exogenous PIM1 renders MPN model cells resistant to ruxolitinib, providing evidence that PIMs could play a role during a JAK2 inhibitor resistant state (Fig. 7 and Supplementary Fig. 7). Thus, combination therapies that include targeting PIMs may increase the efficacy of anti-JAK2 therapy. The JAK2 inhibitors studied to date are type I inhibitors which bind to the kinase when it is in an activated state. Recently investigated type II inhibitors, which bind to kinases in their inactive state, may offer more effective JAK2 inhibition and greater therapeutic efficacy [72, 73]. While type II JAK2 inhibitors may possibly be more effective alone, future combination therapies with such inhibitors may offer a potent anti-MPN therapeutic approach.

In summary, our work utilizing MPN model cells and primary cells from MPN patients demonstrates that targeting PIM kinases may enhance the efficacy of JAK2 inhibitor therapy in MPNs. This may be through enhancing apoptosis of both JAK2 inhibitor sensitive and resistant cells. Clinical testing of PIM inhibitors and JAK2 inhibitors in myelofibrosis was initiated in 2015 (http://ClinicalTrials.gov; NCT02370706).

## MATERIALS AND METHODS

### Ethics statemant

These studies have been conducted in accordance with the ethical standards according to the Declaration of Helsinki and according to national and international guidelines, and have been approved by the authors' institutional review board.

### Cell culture

HEL, SET2, and Uke1, which are JAK2-V617F-positive human myeloid cell lines commonly used to study anti-JAK2/MPN therapeutics, were used in this study. HEL and SET2 cells (a gift from Susumu Kobayashi (Harvard Medical School)) were maintained in RPMI supplemented with 10% FBS and penicillin/streptomycin. Uke1 cells were maintained in RPMI supplemented with 10% FBS, 10% donor equine serum, 1 mM hydrocortisone, and penicillin/streptomycin. BaF3-JAK2-V617F cells were maintained in RPMI supplemented with 10% FBS and penicillin/streptomycin and were previously described [74]. Retrovirus was produced as previously described [74], and infected cells were selected for using hygromycin B. Ruxolitinib resistant/persistent cells were generated by dose escalation as previously described [60]. Dose escalation of ruxolitinib reached 1 μM and cells were maintained in this concentration of ruxolitinib. Experiments using persistent cells were performed using ruxolitinib at this concentration.

### Cell proliferation assays

Relative viable cells were determined by MTS assays using CellTiter 96^®^ AQueous One Solution (Promega Corporation). Samples were read on a Benchmark Plus Microplate Spectrophotometer (Bio-Rad). Data plots and graphs were generated utilizing Prism (GraphPad Software). Combination Indices were calculated using CompuSyn (Combosyn, Inc.). Growth curves were obtained by trypan blue exclusion and data was plotted utilizing Prism (GraphPad Software). The concentration of DMSO (directly added for control samples or as drug solvent for drug treated samples) was kept constant (0.1%) for each treatment for all experiments.

### Annexin V staining

Cells were analyzed using the FITC Annexin V detection kit (#556547, BD Pharmingen) and flow cytometry. Briefly, cells (1 × 10^6^) were washed with PBS and resuspended in 100 uL of 1X Annexin V Binding Buffer containing 4.6 uL of staining solution (1.6 uL of 50 ug/mL propidium iodide and 3 uL Annexin V-FITC). Cells were incubated for 15 minutes at room temperature, followed by addition of 300 uL of 1X Annexin V Binding Buffer. Samples were analyzed by flow cytometry.

### Inhibitors, antibodies, and immunoblotting

SGI-1776, ruxolitinib, and bortezomib were obtained from Selleck Chemicals. AZD1208 was obtained from AstraZeneca, Inc. All drugs were solubilized in DMSO and stored at −20 or −80°C. Antibodies used in this study were: phospho (P)-p70S6K (T389) (#9234S); P-BAD (S112) (#9296); P-4EBP1 (T37/46) (#2855); P-S6 (S235/236) (#4858); BAD (#9329); hu-cl-PARP (#5625); mm-cl-PARP (#9544); GAPDH (#5174) (Cell Signaling Technology); Tubulin (#SC-5286); (Santa Cruz Biotechnology); and actin (#A5316) (Sigma-Aldrich). Antibody for mouse MDM2 was a gift from Jiandong Chen (Moffitt Cancer Center) and was previously described [75]. For immunoblotting, protein concentration was determined using Pierce™ BCA Protein Assay kit (Thermo Scientific) and a Benchmark Plus Microplate Spectrophotometer (Bio-Rad), and immunoblots were performed by standard SDS-PAGE. Horseradish peroxidase-conjugated secondary antibodies were from Thermo Scientific. Blots were developed using chemillumination detection reagents (Thermo Scientific).

### Colony formation assay

Peripheral blood was obtained from patients consented through the Moffitt Cancer Center Total Cancer Care protocol (MCC 14690/ Liberty IRB #12.11.0023) and approved by the Moffitt Cancer Center Scientific Review committee. Blood was treated with HetaSep™ (STEMCELL Technologies, Inc.) to remove the majority of red blood cells. Peripheral blood mononuclear cells (PBMCs) were isolated by ficoll separation. PBMCs (1 - 4 × 10^5^) were then plated in 1 mL of methylcellulose medium containing rhSCF, rhIL-3, and rhGM-CSF (MethoCult™ #H4534; STEMCELL Technologies, Inc.). All drug treated samples contained 0.1% DMSO as the final concentration. For healthy controls, 3 U/mL Epo was added. Cells were incubated at 37° C with 5% CO_2_ and erythroid colonies were enumerated after 12–14 days. Data graphs and statistics were generated utilizing Prism (GraphPad Software).

### PIM1 exogenous expression

The cDNAs for human PIM1 L and S were obtained from Dr. Yun Qiu (University of Maryland School of Medicine), and contained a FLAG sequence at the amino terminus. These FLAG-PIM1 cDNAs were subcloned into pBABE-Hygro [76] utilizing In-Fusion^®^ technology (Clontech^®^). Sequences of the sub-cloned cDNAs were confirmed by bi-directional sequencing.

## SUPPLEMENTARY FIGURES


