# Supplementary material for: The PIM inhibitor AZD1208 synergizes with ruxolitinib to induce apoptosis of ruxolitinib sensitive and resistant JAK2-V617F-driven cells and inhibit colony formation of primary MPN cells
Source: Oncotarget. 2015 Oct 12;6(37):40141–57. doi: 10.18632/oncotarget.5653 (PMC4741885; doi:10.18632/oncotarget.5653)
Supplement: Supplementary file 1 [file oncotarget-06-40141-s001.pdf]

SUPPLEMENTARY FIGURES

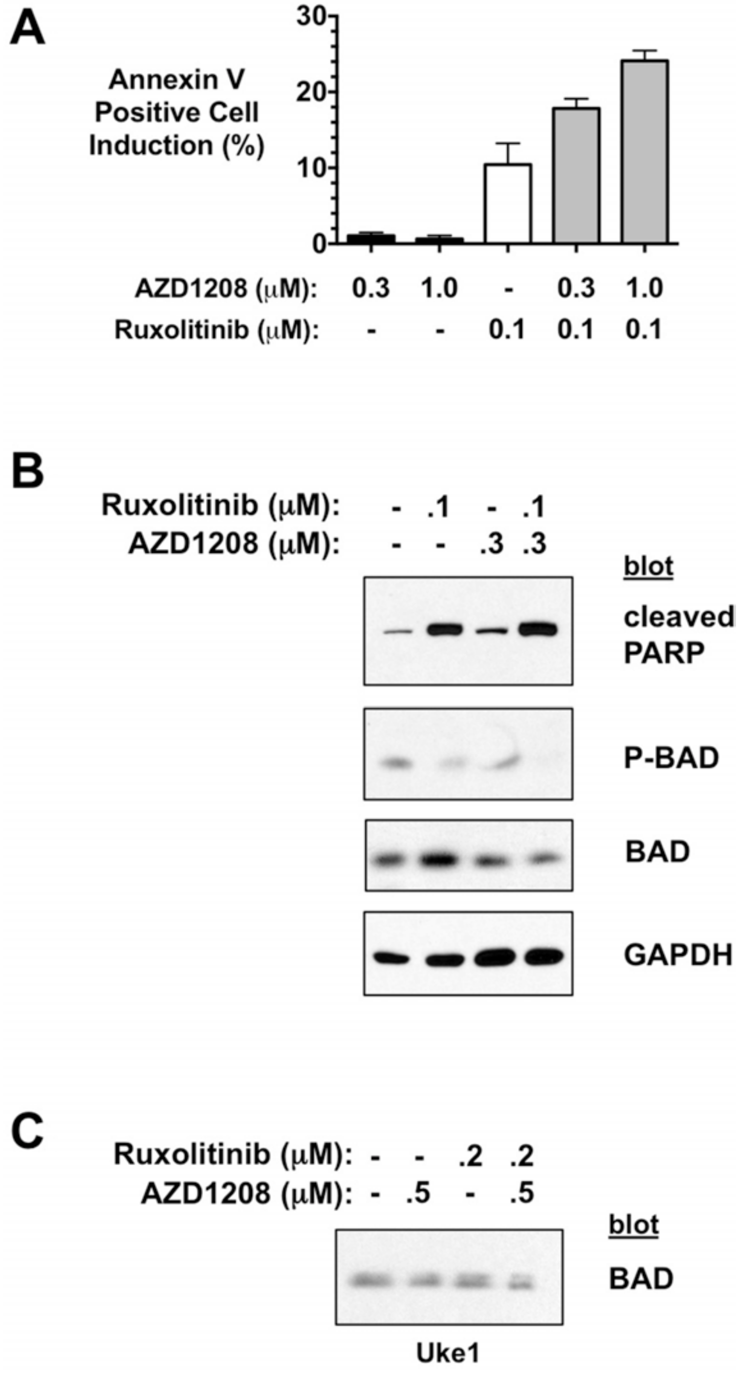

Supplementary Figure S1: AZD1208 enhances SET2 cell apoptosis induced by ruxolitinib. A. SET2 cells were treated with DMSO, AZD1208 (0.3 µM and 1.0 µM), ruxolitinib (0.1 µM), and AZD1208 plus ruxolitinib in combination. Annexin V binding was determined by flow cytometry after 96 hours of treatment. Data is represented as the increase in the percent of annexin V positive cells compared to DMSO-treated cells. Error bars indicate standard deviation of samples treated in triplicate. **B.** SET2 cells were treated with DMSO, AZD1208 (0.3 µM), ruxolitinib (0.1 µM), and AZD1208 and ruxolitinib in combination. Cell lysates were prepared after 24 hours of treatment and immunoblots were performed for cleaved PARP, P-BAD (Ser-112), BAD, and GAPDH as a loading control, as indicated. **C.** Uke1 cells were treated with DMSO (-), AZD1208 (0.5 uM), ruxolitinib (0.2 uM), and AZD1208 and ruxolitinib in combination. Cell lysates were prepared after 24 hours of treatment and an immunoblot was performed for BAD, demonstrating drug treatment does not alter BAD expression.

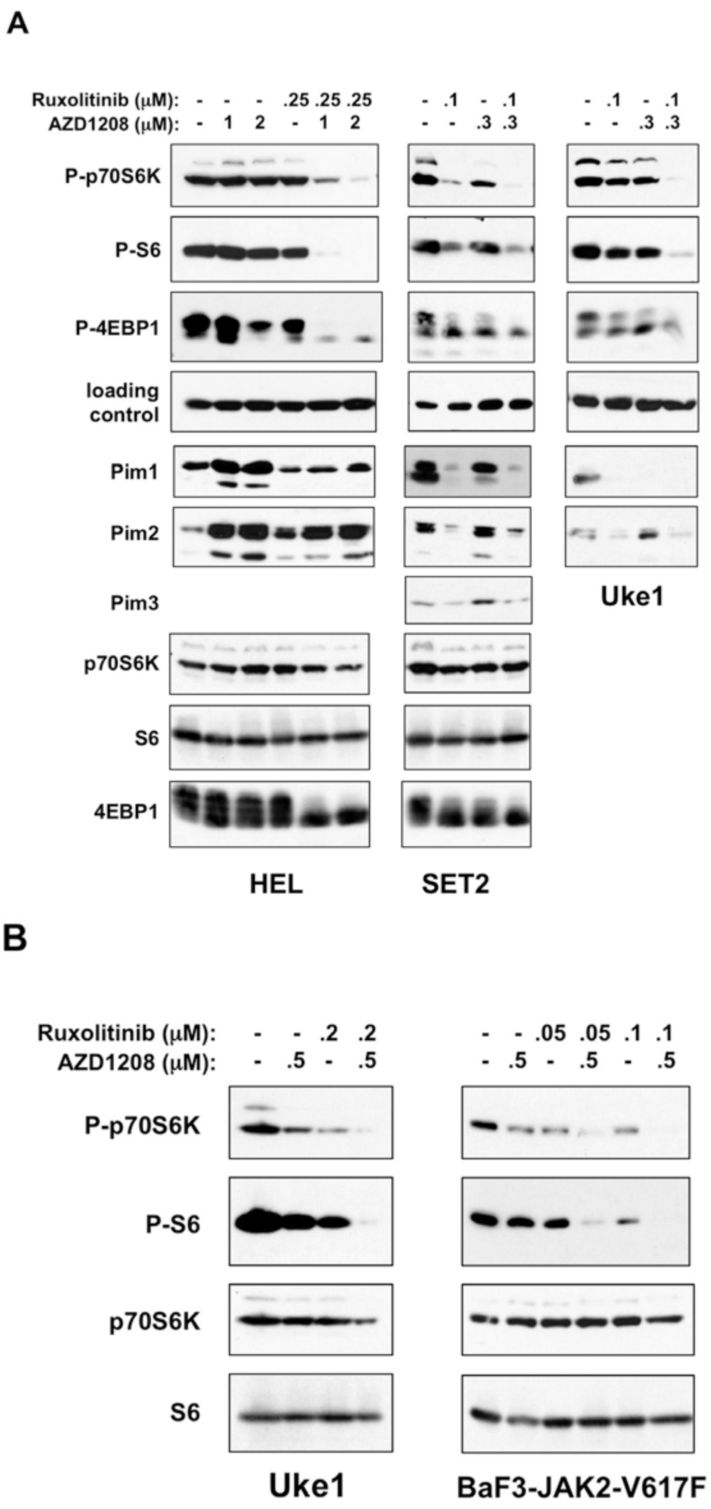

Supplementary Figure S2: AZD1208 and ruxolitinib suppress downstream signaling of the mTOR pathway. A. The top part of this figure is the same as in Figure 5 for easy reference. HEL, Set2, and Uke1 cells were treated with DMSO (-) or the indicated amounts of AZD1208 and ruxolitinib, alone and in combination. Lysates were prepared following 24 hours for HEL cells, and 4 hours for SET2 and Uke1 cells. Lysates were analyzed by immunoblotting for P-p70S6K (T389), P-S6 (S235/236), P-4EBP1 (T37/46), PIM1, PIM2, PIM3, p70S6K, S6k, and 4EBP1 as indicated. Loading controls were tubulin (for HEL and Uke1) and GAPDH (for Set2). Note: the loading control blot for HEL cells in this figure is the same as shown in Figure 3, as the same lysates were analyzed in each figure. **B.** Uke1 and BaF3-JAK2-V617F cells were treated with DMSO (-) or the indicated amounts of AZD1208 and ruxolitinib, alone and in combination. Lysates were prepared following 24 hours for Uke1 cells and 48 hours for BaF3-JAK2-V617F cells. Lysates were analyzed by immunoblotting for P-p70S6K (T389), P-S6 (S235/236), p70S6K, and S6K as indicated.

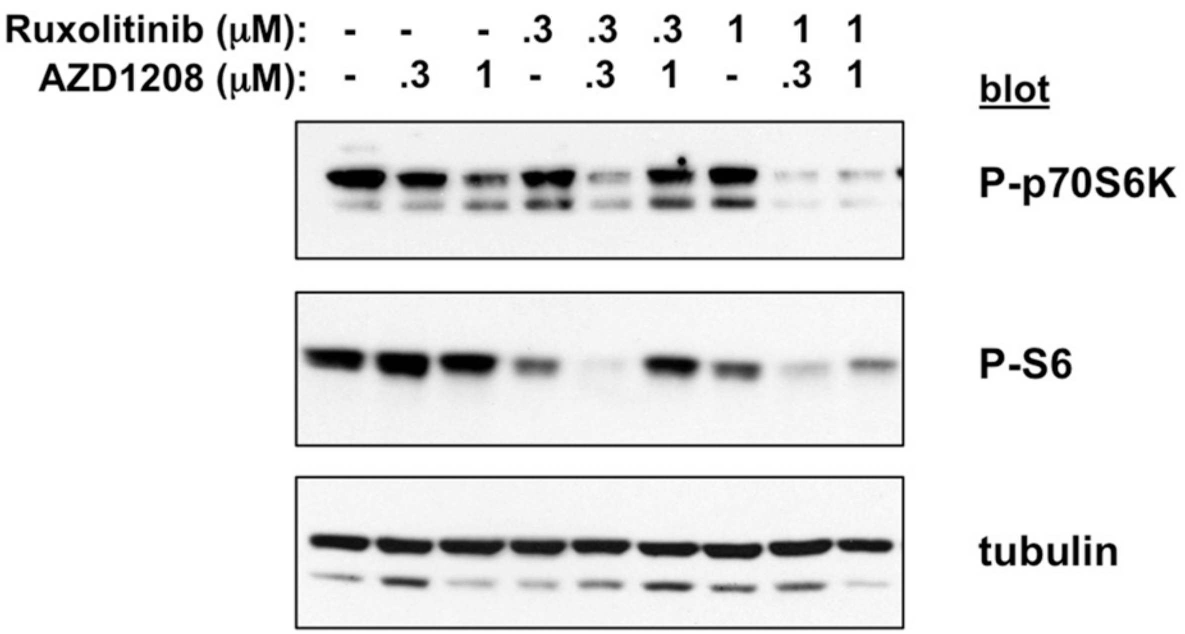

Supplementary Figure S3: AZD1208 and ruxolitinib suppress signaling to phosho-p70S6K and phospho-S6 in primary MPN patient granulocytes. Granulocytes from a JAK2-V617F-positive PV patient were treated with DMSO (-) or the indicated amounts of AZD1208 and ruxolitinib, alone and in combination for five hours. Lysates were analyzed by immunoblotting for P-p70S6K (T389), P-S6 (S235/236), and tubulin as a loading control.

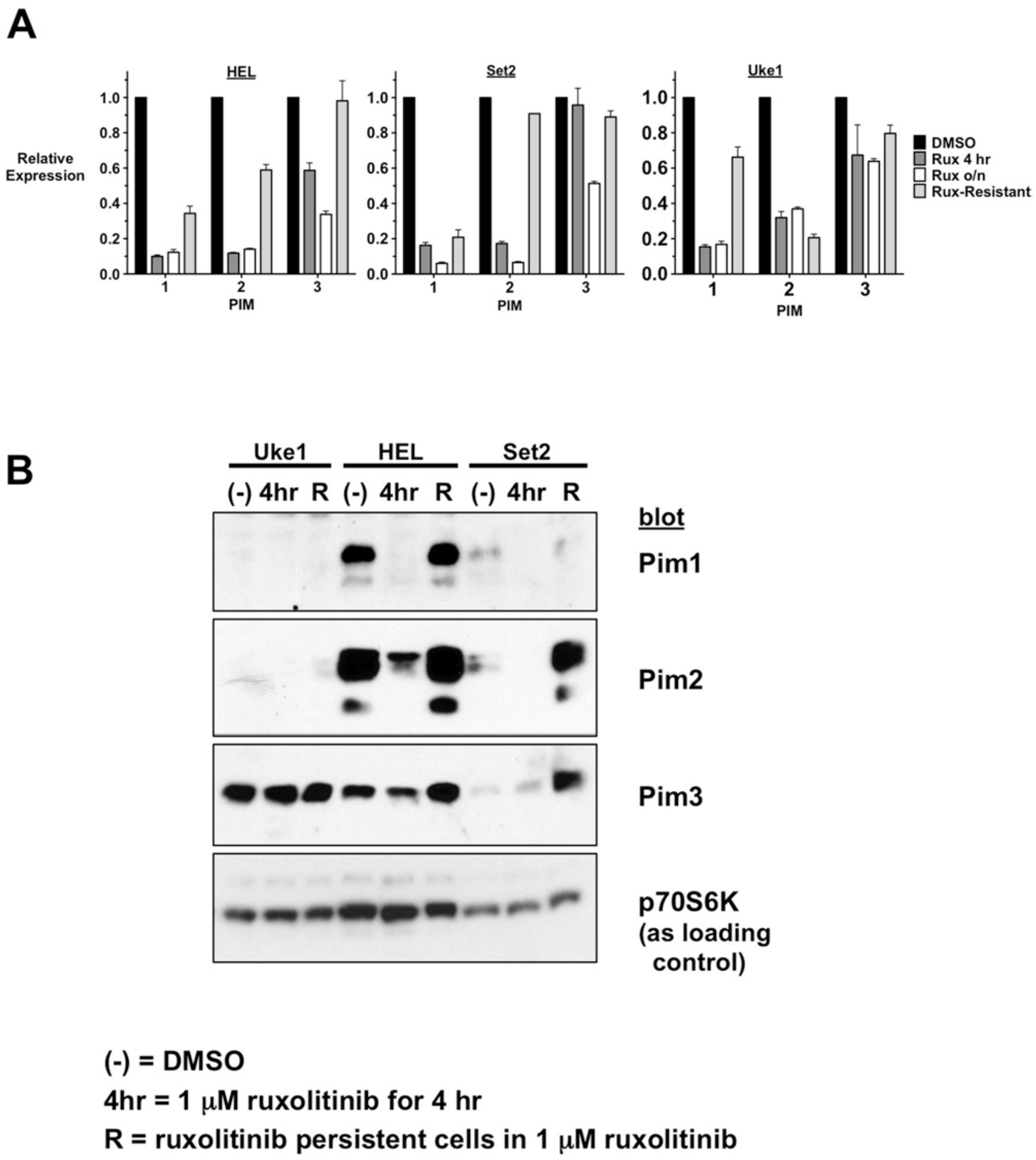

Supplementary Figure S4: Expression of PIM mRNA following ruxolitinib treatment and in ruxolitinib persistent cells. A. HEL, SET2, and Uke1 cells were treated with DMSO overnight and with ruxolitinib (1 uM) for 4 or 20 hours (o/n). Quantitative-RT-PCR was performed on these samples, as well as ruxolitinib persistent cells, which are resistant to ruxolitinib, to detect mRNA expression for PIM1, PIM2, and PIM3. Total RNA obtained from these ruxolitinib-sensitive treated cells as well as ruxolitinib persistent cells was subjected to *PIM1, 2,* and *3* transcript specific qRT-PCR. Expression of *PIM1, 2,* and *3* was determined relative to the corresponding DMSO-treated cells and normalized with 18S expression within each sample (ΔΔct). **B.** HEL, Set2, and Uke1 cells were treated with DMSO (-) or 1 μM ruxolitinib for 4 hr. Lysates from these and ruxolitinib persistent cells growing in 1 μM ruxolitinib were blotted for PIM1, PIM2, PIM3, and p70S6K (as a loading control).

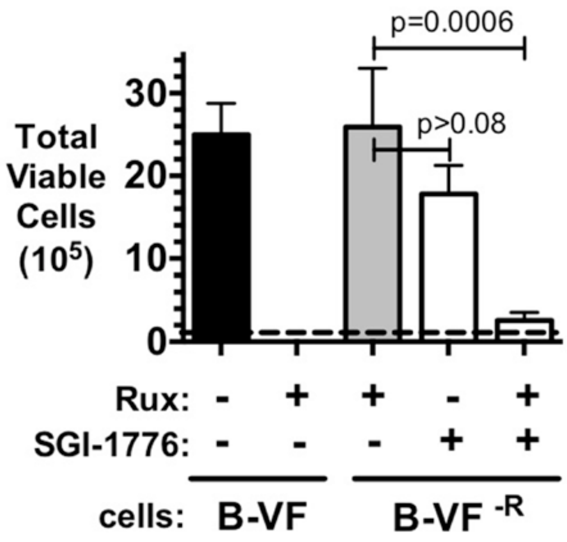

Supplementary Figure S5: BAF3-JAK2-V617F cells that persistently grow in ruxolitinib are sensitive to the combination of ruxolitinib and SGI-1776. BaF3-JAK2-V617F (B-VF) cells or the same cells rendered persistent to the JAK2 inhibitor ruxolitinib ($B\text{-}VF^{-R}$), were cultured in the presence or absence of ruxolitinib (0.5 μM) (Rux) or the PIM inhibitor SGI-1776 (1 μM), as indicated, for three days. Total viable cells were determined by trypan blue exclusion. The dashed line indicates the starting number of cells (1×10^5), error bars indicate s.d., and *p*-value was calculated by *t*-test.

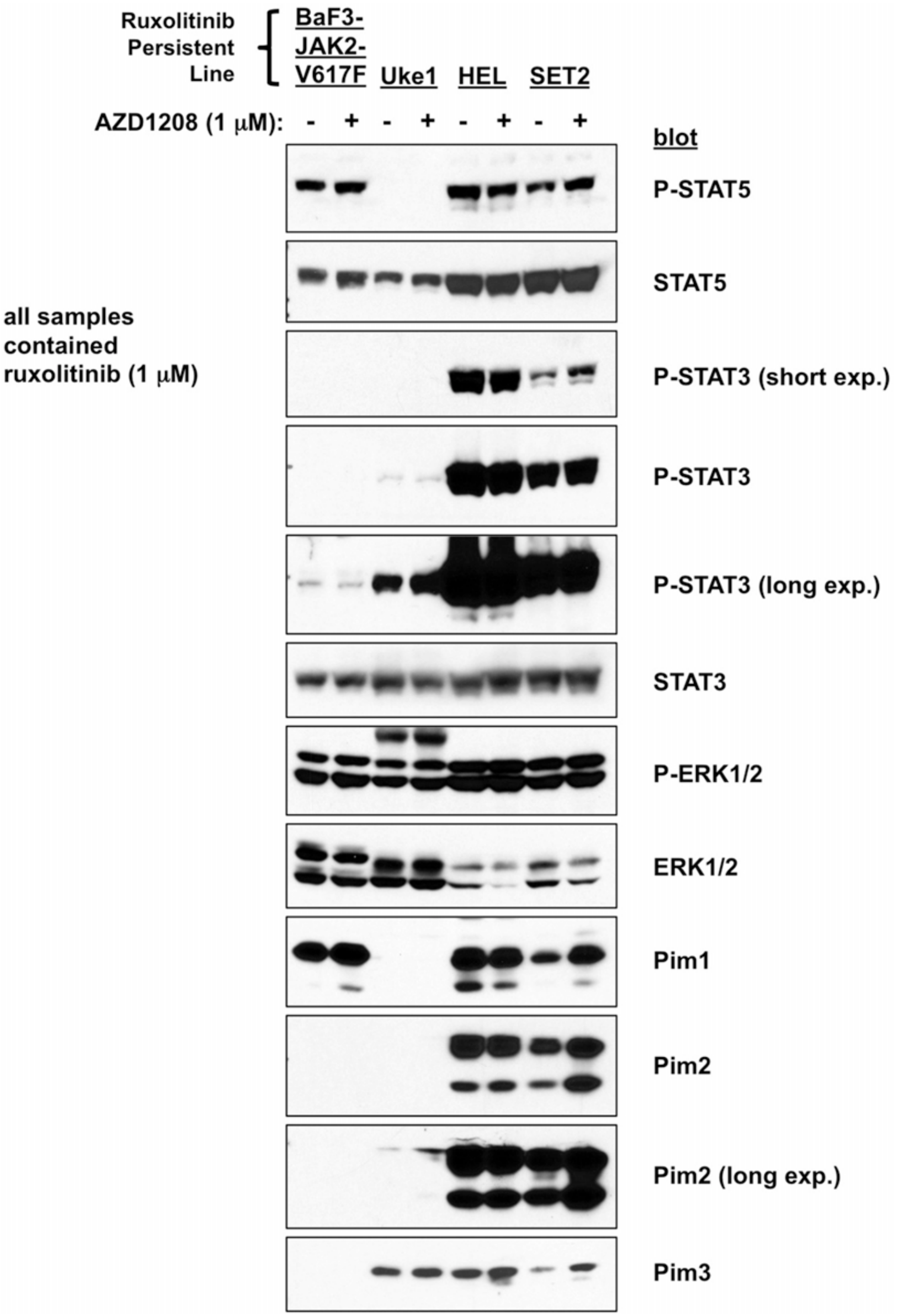

Supplementary Figure S6: AZD1208 treatment of ruxolitinib persistent cells does not affect activation of downstream effectors of JAK2 signaling. JAK2 inhibitor persistent BAF3-JAK2-V617F, Uke1, HEL, and SET2 cells were incubated overnight with DMSO (-) or 1 μM AZD1208 (+). Lysates were analyzed by immunoblotting for the total and phosphorylated/active forms of STAT5, STAT3, and ERK1/2, as well as for PIM1, PIM2, and PIM3. For some blots different exposures are shown for more complete interpretation. Note: the PIM2 antibody used does not recognize mouse PIM2 (BaF3 cells) and the epitope for the PIM3 antibody does differ between human and mouse PIM3. Antibodies used were P-STAT5 (Y694) (#611961, BD Transduction Lab), P-STAT3 (Y705) (#9138), P-ERK (T202/Y204) (#4370), PIM2 (#4730), and PIM3 (#4165) (Cell Signaling Technology®); PIM1 (#sc-13513), STAT5 (#sc-483), and STAT3 (#sc-835) (Santa Cruz Biotechnology).

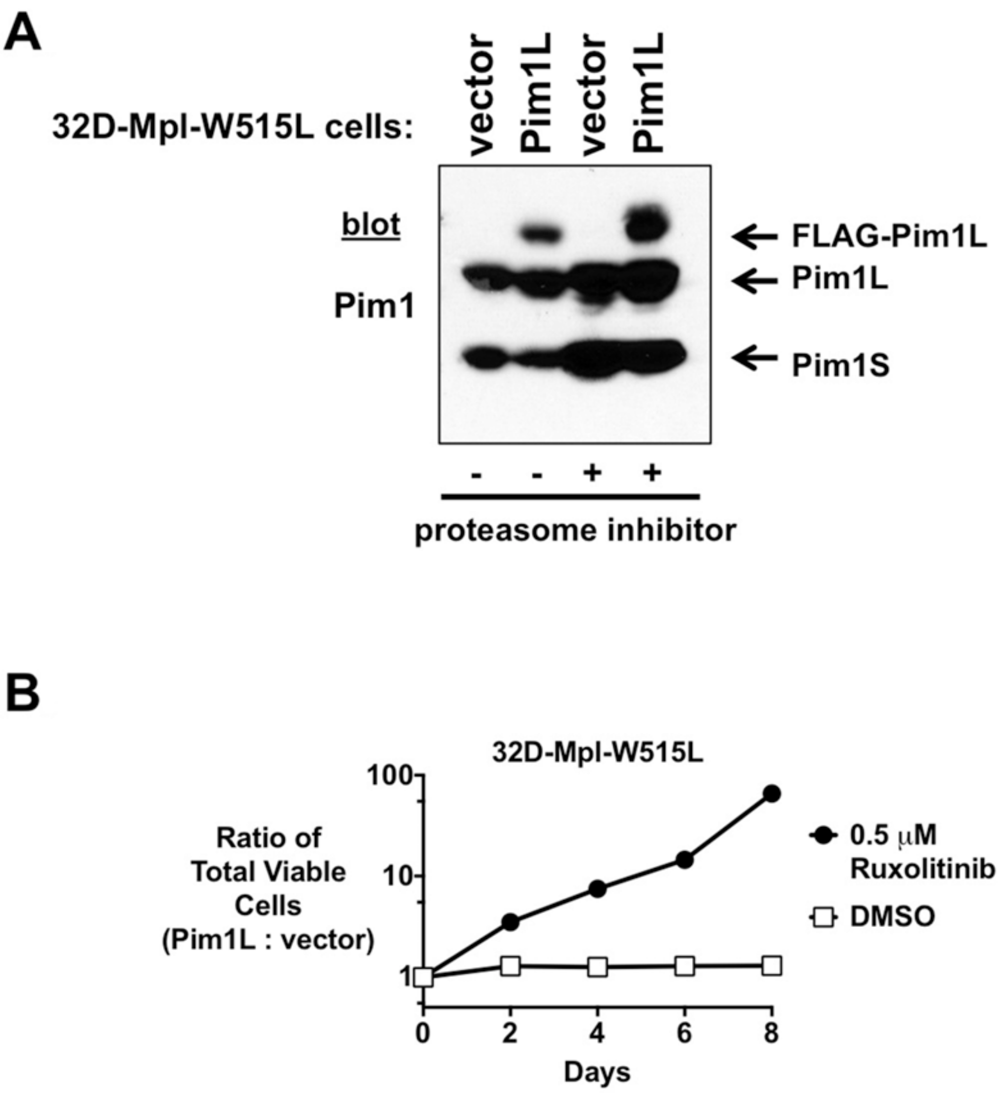

Supplementary Figure S7: Exogenous expression of PIM1 induces ruxolitinib resistance in 32D-Mpl-W515L cells. A. PIM1L was expressed from a viral promoter in 32D cells that were transformed by expression of Mpl-W515L. Cell lysates were immunoblotted for PIM1. The proteasome inhibitor bortezomib was utilized, as indicated, to stabilize PIM1 expression for easier detection. The exogenous PIM1L protein was FLAG-tagged thus increasing its molecular weight and slowing its mobility in SDS-PAGE compared to endogenous PIM1L. Mobility of endogenous and exogenous PIM1 proteins are indicated with arrows. **B.** The PIM1L-expressing cells from A., as well as empty vector control cells, were cultured with DMSO or ruxolitinib (0.5 μM) and total viable cells were determined over time by trypan blue exclusion. The ratio of total viable PIM1L-expressing cells to total viable vector cells is plotted over time. The horizontal line for the DMSO-treated cells indicates equal rate of growth for vector and PIM1L-expressing 32D-Mpl-W515L cells. The increasing slope of the line for the ruxolitinib-treated cells indicates that PIM1L-expressing cells increased in number over time while the vector control cells did not, thus representing induction of drug resistance by exogenous PIM1L expression.
